# Spontaneous Rupture of Renal Metastasis from Hepatocellular Carcinoma

**DOI:** 10.1155/2017/8607061

**Published:** 2017-05-22

**Authors:** Osamu Kinoshita, Yusuke Ichijo, Masayuki Yoneda, Atsushi Ikai, Tetsuro Yamashita

**Affiliations:** ^1^Department of Surgery, Maizuru Medical Center, Kyoto, Japan; ^2^Department of Radiology, Kyoto Prefectural University of Medicine, Kyoto, Japan

## Abstract

We report a rare life-threatening case of spontaneous rupture of renal metastasis from hepatocellular carcinoma (HCC) that was managed by emergent transcatheter arterial embolization (TAE). A 76-year-old woman diagnosed with HCC presented with acute back pain in her right side and was transferred to our hospital. Initial enhanced computed tomography revealed retroperitoneal hemorrhage from the right kidney, which was retrospectively diagnosed as a spontaneous rupture of the metastatic renal tumor from the primary HCC. Detailed examination identified an active retroperitoneal hemorrhage from the lesion and the patient's condition became hemodynamically unstable; hence emergent TAE was performed. The hospitalization period after the TAE was uneventful and sorafenib was subsequently administered. Unfortunately, two months after the TAE, the tumor locally progressed within the retroperitoneal space. Tumors were controlled by repeated TAE as the patient did not want to undergo a nephrectomy. Consequently, she survived for more than one year after emergent TAE, exhibiting low levels of tumor marker. After rupture of the metastatic renal HCC, tumors were expected to progress into the retroperitoneal space, and nephrectomy was the next possible radical treatment to offer the best chance of long-term disease control.

## 1. Introduction

Hepatocellular carcinoma (HCC) is the third-leading cause of cancer-related death worldwide [1]. Despite a decline in its mortality rate, HCC remains the fourth-leading cause of cancer-related death in Japan [2]. Following liver resection to treat HCC, its recurrence pattern is mostly intrahepatic, and its high recurrence ratio generally makes treatment difficult. However, establishing treatment strategies for intrahepatic HCC offers long-term disease control [3]. Thanks to improvements in diagnostic imaging modalities, HCC cases with extrahepatic metastasis are being diagnosed more frequently. Reports suggest that extrahepatic HCC metastasis is detected in 8.2%–15.2% of patients who initially present with intrahepatic disease [4, 5], and others have documented it in 63% of a total 98 autopsy cases [6]. Regarding sites of extrahepatic HCC metastasis, many researchers have reported rare locations of metastasis; however, very few reports describe renal metastasis from HCC. Moreover, spontaneous rupture of renal metastasis remains a rarer event still. We herein present a life-threatening case of spontaneous rupture of a renal metastatic tumor from HCC and management by transcatheter arterial embolization (TAE) and report the posttherapeutic progress of the tumor.

## 2. Case Presentation

**Figure 1:**
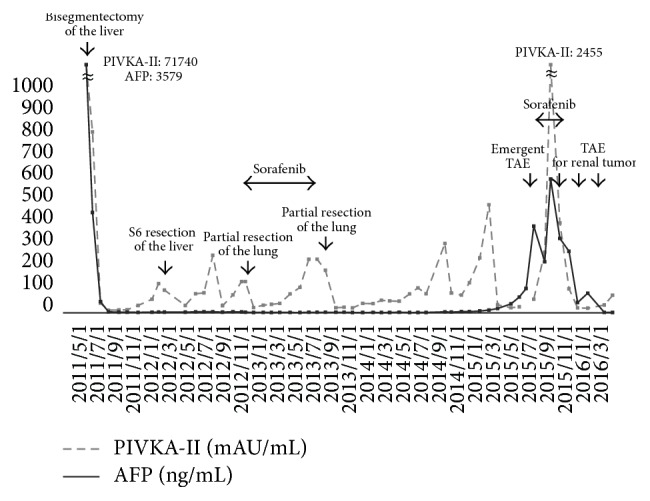
Time course of tumor marker during treatment period.

A 76-year-old woman presented with acute back pain in her right side and was transferred to Maizuru Medical Center in June 2015. She was diagnosed with HCC, which had arisen from hepatitis C infection, and Child-Pugh Class A cirrhosis. She underwent a central bisegmentectomy of the liver in June 2011, S6 partial liver resection in February 2012, and two partial resections of the lung in November 2012 and August 2013. She was required to undergo follow-up surveillance every three months. As shown in Figure 1, the aforementioned surveillance indicated elevated levels of serum protein induced by vitamin K absence II (PIVKA-II) and alpha-fetoprotein (AFP); however, the intrahepatic space-occupying lesion was not identified by the follow-up enhanced computed tomography (CT) and ultrasonography.

**Figure 2:**
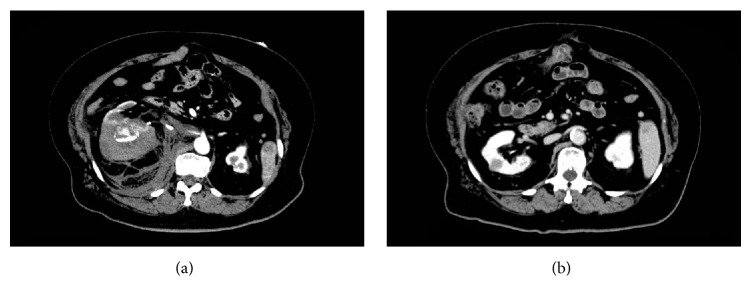
Computed tomography (CT) findings. (a) The initial enhanced CT (arterial phase), depicting massive retroperitoneal bleeding. (b) The previous CT (late portal phase), performed one year before the emergent admission, depicting the renal tumor mimicking a simple renal cyst.

On admission, the laboratory data indicated severe anemia, as shown in Table 1. The initial enhanced CT revealed an active retroperitoneal hemorrhage surrounding the right renal tumor, measuring 80 × 60 mm (maximum dimensions) (Figure 2(a)). When retrospectively reviewed, a prior enhanced CT revealed that the right renal tumor mimicked a simple renal cyst, which exhibited low density by plain examination and no enhancing effect from the contrast agent (Figure 2(b)). However, the size of the tumor gradually increased over the follow-up period, and it was possibly recognized as a metastatic renal tumor from the known HCC. Therefore, we provisionally diagnosed that the retroperitoneal hemorrhage was a result of the spontaneous tumor rupture.

**Figure 3:**
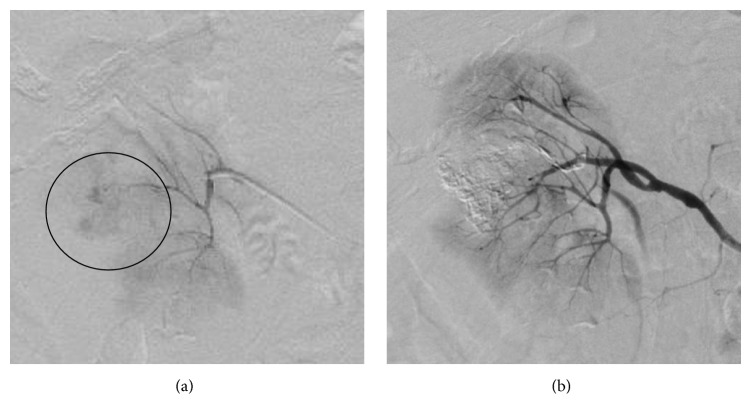
Right renal arteriography. (a) The tumor was fed by the dorsal branch of the renal artery and extravasation from the tumor. (b) Hemorrhage disappeared after the TAE.

**Figure 4:**
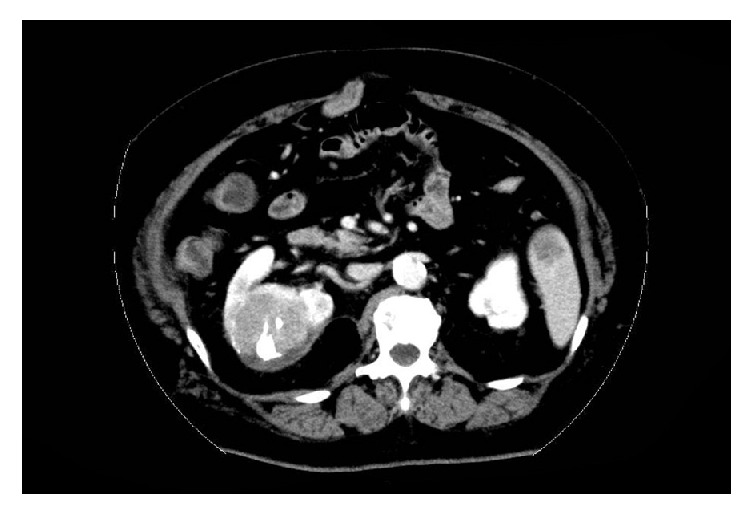
Follow-up CT, performed in September 2015. Marked early enhancement on arterial phase and wash-out on late portal phase indicate growing residual viable lesion.

On admission, the blood pressure of the patient was 90/64 mmHg, while her heart rate was 109 bpm with a regular rhythm, respiratory rate was 23 breaths/min, and peripheral oxygen saturation was 100%. Because of hemodynamic instability and rapidly progressing anemia (34.2 g/dL on admission and 27.3 g/dL three hours after admission), emergent renal arteriography was performed (Figure 3), and hemostasis was subsequently carried out by TAE using 40% N-butyl-2-cyanoacrylate (NBCA) administered selectively to the right renal tumor. The blood flow of the nontargeted renal arterial branch after TAE completion was satisfactory, and posttherapeutic renal function was maintained within normal ranges with no complications. The hospitalization period after the TAE was uneventful, and the patient was discharged on posttherapeutic day seven. Administration of sorafenib was commenced in the outpatient clinic; however, the tumor progressed into the retroperitoneal space two months after the emergent TAE (Figure 4). Because of the absence of intrahepatic HCC, we proposed that the patient should undergo a right nephrectomy, but she did not consent to the procedure. Thus, the renal tumor was controlled by repeated TAE, and the patient expressed low levels of serum PIVKA-II and AFP for at least one year after the emergent TAE.

## 3. Discussion

In agreement with the Japanese analysis performed on autopsy specimens from HCC patients, the occurrence of extrahepatic HCC metastasis was retrospectively calculated as 45.6% in lung, 30.7% in lymph node, 17.8% in peritoneum, 14.5% in bone, and 11.1% in adrenal gland [6]. A recent report showed that, of 47 cases initially presenting with extrahepatic HCC with a prior diagnosis of primary intrahepatic HCC, 17 cases (36%) of extrahepatic HCC occurred in bone, nine (19%) in lymph node, seven (14%) in soft tissue, and seven (14%) in omentum [7]. However, because of its rarity, the frequency of renal metastasis from HCC remains unclear in the literature. A PubMed search was performed to identify case reports published over the past three decades, using the keywords “hepatocellular carcinoma (HCC)” and “renal metastasis”; additionally, further relevant articles were identified by a manual search of the references of the key articles. Consequently, six cases were identified [8–13], and, of those, only one report described “spontaneous rupture” of the tumor [10]. Accordingly, to the best of our knowledge, the present case is the second report of a spontaneous rupture of a renal metastatic tumor from HCC.

One of the life-threatening complications of HCC is spontaneous tumor rupture, and previous literature has reported the considerable mortality rates associated with this [14]. Although TAE is well-recognized as a treatment option for intrahepatic HCC rupture [3, 15], little data is available for rupture of metastatic renal tumors from extrahepatic HCC. Similar to our case, Mezawa et al. [10] reported a case of metastatic renal tumor rupture from HCC, focusing particularly on TAE management. However, because the patient in the previous report died from brain metastasis three months after the TAE, detailed data for the possible progression of the renal tumor after TAE was not available. We believe that our case is complementary to the previous literature and contributes to the further development of disease management for extrahepatic HCC.

In the present case, despite the administration of sorafenib, the tumor progressed into the retroperitoneal space two months after the emergent TAE. Several treatment strategies for intrahepatic HCC have been proposed [3]; however, those for extrahepatic HCC, especially for renal HCC metastasis, have not reached a consensus. Similar to renal metastasis, some researchers have reported that surgical resection of metastatic HCC in the adrenal gland resulted in long-term survival [16]. Therefore, although the patient in the current study did not wish to have a nephrectomy, we believe this approach would have offered the best chance of long-term disease control.

A limitation of the diagnosis of our case was that histology from the right renal tumor was not obtained over the treatment course, and the tumor was not pathologically proven as metastasis from HCC. However, the fact that the TAE for the renal tumor led to normalization of PIVKA-II and AFP levels strongly suggested that the tumor was an extrahepatic HCC lesion (Figure 1). In addition, although the renal tumor resembled a simple renal cyst at initial diagnosis, the sequential imaging findings after TAE were also compatible with that of recurrent metastatic HCC.

In summary, we examined a rare case of spontaneous rupture of renal metastasis from HCC, with the resulting retroperitoneal bleeding successfully controlled by emergent TAE. After the rupture of a metastatic renal HCC, tumors would be expected to progress into the retroperitoneal space, as demonstrated in the present case; nephrectomy is the next possible treatment option to offer the best chance of long-term disease control.

## Figures and Tables

**Table 1:** Laboratory data on admission.

Indicators	Value
White blood cell	5200/*μ*L
Hemoglobin	11.3 g/dL
Platelet	103 × 10^3^/*μ*L
Prothrombin time	96%
Activated partial thromboplastin time	22.6 s
Aspartate aminotransferase	26 U/L
Alanine aminotransferase	20 U/L
Lactate dehydrogenase	244 U/L
Alkaline phosphatase	280 U/L
Total bilirubin	0.7 mg/dL
Total protein	6.9 g/dL
Albumin	3.9 g/dL
Blood urea nitrogen	25.6 mg/dL
Creatinine	1.05 mg/dL
Uric acid	7.3 mg/dL
Na	139 mEq/L
K	6.9 mEq/L
Cl	104 mEq/L
Total cholesterol	169 mg/dL
C-reactive protein	0.11 mg/dL
Protein induced by vitamin K absence II	61 mAU/mL
*α*-Fetoprotein	384 ng/mL
